# 

**Published:** 1868-04

**Authors:** 


### IOWA MEDICAL JOURNAL,

A BI-MONTHLY, OF 32 PAGES.

*Price, Two Dollars per Annum, Payable in Advance.*

*Gentlemen of the Profession:*

AFTER a suspension of several years, the publication of THE IOWA MEDICAL JOURNAL has again been resumed. The first number of volume five was issued for November and December, 1867. The time of issue of the second number having arrived, the editor, after consulation with the Committee on Publication for the State Medical Society, in order that the proceedings of the Society might be placed in the hands of its members at an early day, thought it best to print a double number, which explains the size of the present issue.

The encouragement received by the editor, thus far, has not been of a substantial character. Complimentary letters will not pay the printer, nor will they furnish food for paying subscribers. We have numerous orders for THE JOURNAL, but thus far few paying subscribers, as may be seen by referring to the list of honor. We shall supply orders from the first issue, trusting that promises will be promptly complied with. Give me the proper encouragement, gentlemen, and I will endeavor to make THE IOWA MEDICAL JOURNAL a welcome visitor to every subscriber. All letters and communications for THE JOURNAL may be addressed to

J. C. HUGHES, M. D.,
*Keokuk, Iowa.*

